# Supplementary material for: Association of birth and childhood weight with risk of chronic diseases and multimorbidity in adulthood
Source: Commun Med (Lond). 2023 Jul 31;3:105. doi: 10.1038/s43856-023-00335-4 (PMC10390459; doi:10.1038/s43856-023-00335-4)
Supplement: Supplementary file 2 — Supplementary information [file 43856_2023_335_MOESM2_ESM.pdf]

Association of birth and childhood weight with risk of chronic diseases and multimorbidity in adulthood

Yue Zhang[1,2#], Yaguan Zhou[1,2#], Yangyang Cheng[1,2], Rodrigo M Carrillo-Larco[3], Muhammad Fawad[1,2], Shu Chen[4,5], Xiaolin Xu[1,2,6*]

1. School of Public Health and The Second Affiliated Hospital, Zhejiang University School of Medicine, Hangzhou, Zhejiang, China
2. Key Laboratory of Intelligent Preventive Medicine of Zhejiang Province, Hangzhou, Zhejiang, China
3. Hubert Department of Global Health, Rollins School of Public Health, Emory University, Atlanta, GA, USA
4. Australian Research Council Centre of Excellence in Population Ageing Research (CEPAR), University of New South Wales, Sydney, Australia.
5. School of Risk & Actuarial Studies, University of New South Wales, Sydney, Australia.
6. School of Public Health, Faculty of Medicine, The University of Queensland, Brisbane, Australia

# These authors contributed equally.

*Corresponding Author:

Xiaolin Xu, PhD, School of Public Health and The Second Affiliated Hospital, Zhejiang University School of Medicine, Yuhangtang Road 866, Hangzhou, Zhejiang 310058, China. Email: xiaolin.xu@zju.edu.cn

Supplementary Information

Contents

Supplementary Tables 3

Supplementary Table S1. List of the 38 chronic conditions included in the definition of multimorbidity. 3

Supplementary Table S2. Distribution of family history of major diseases. 4

Supplementary Table S3. Comparison of characteristics of participants who were excluded in the analyses versus included individuals (n = 502,490). 5

Supplementary Table S4. Factor loadings of each chronic condition for the six multimorbidity patterns. 6

Supplementary Table S5. Estimated ORs (95% CIs) for the associations of birth weight and childhood body size with multimorbidity patterns. 7

Supplementary Table S6. Sensitivity analysis of the association between birth weight categories and the incidence of multimorbidity. 8

Supplementary Table S7. Sensitivity analysis of the association of birth weight and childhood body size with the incidence of multimorbidity by excluding individuals with missing information on covariates (n=139,546). 8

Supplementary Table S8. Sensitivity analysis of the association of birth weight and childhood body size with the incidence of multimorbidity by imputing missing covariates with multiple imputation. 8

Supplementary Table S9. Sensitivity analysis of the association of birth weight, childhood body size, and their changes with the incidence of multimorbidity by additionally adjusting family history of major chronic diseases. 9

Supplementary Figures 10

Supplementary Figure S1. Flow chart of the selection process. 10

Supplementary Figure S2. Restricted cubic spline models for the relationship of birth weight with the incidence of part of individual chronic conditions. 11

Supplementary Figure S3. Restricted cubic spline models for the relationship of birth weight with the incidence of part of individual chronic conditions. 12

Supplementary Figure S4. Restricted cubic spline models for the relationship of birth weight with the incidence of the remaining individual chronic conditions. 13

Supplementary Figure S5. Subgroup analysis of the association of birth weight and childhood body size with the incidence of multimorbidity. 14

Supplementary Figure S6. Association of birth weight and childhood body size with the incidence of multimorbidity stratified by family history of major disease. 15

Supplementary Tables

Supplementary Table S1. List of the 38 chronic conditions included in the definition of multimorbidity.

NO.	Chronic conditions
1	Atrial fibrillation
2	Angina
3	Anxiety
4	Asthma
5	Bronchiectasis
6	Cancer
7	Cirrhosis
8	Chronic kidney disease
9	Chronic obstructive pulmonary disease
10	Dementia
11	Depression
12	Diabetes
13	Eczema or dermatitis
14	Epilepsy
15	Glaucoma
16	Hepatitis
17	Heart failure
18	Hypertension
19	Irritable bowel syndrome
20	Inflammatory bowel disease
21	Myocardial infarction
22	Migraine
23	Multiple sclerosis
24	Osteoporosis
25	Parkinson's disease
26	Prostate problem
27	Peripheral vascular disease
28	Rheumatoid arthritis
29	Schizophrenia
30	Chronic sinusitis
31	Stroke
32	Thyroid problem
33	Dyspepsia
34	Constipation
35	Hearing loss
36	Diverticular disease of intestine
37	Endometriosis
38	Meniere's disease

Supplementary Table S2. Distribution of family history of major diseases.

	Overall	Birth weight			P value	Childhood body size			P value
		Normal birth weight	Low birth weight	High birth weight		Average	Thinner	Plumper	
**N**	246,495	190,359	21,626	34,510		125,803	79,700	40,992	
Heart disease	110,381 (44.8)	84,218 (44.2)	10,551 (48.8)	15,612 (45.2)	**<0.001**	55,041 (43.8)	35,751 (44.9)	19,589 (47.8)	**<0.001**
Stroke	65,045 (26.4)	49,392 (26.0)	6,473 (29.9)	9,180 (26.6)	**<0.001**	32,576 (25.9)	21,198 (26.6)	11,271 (27.5)	**<0.001**
Cancer	86,291 (35.0)	65,605 (34.5)	7,875 (36.4)	12,811 (37.1)	**<0.001**	43,786 (34.8)	28,058 (35.2)	14,447 (35.2)	0.099
Chronic bronchitis/emphysema	38,726 (15.7)	28,862 (15.2)	4,380 (20.3)	5,484 (15.9)	**<0.001**	18,703 (14.9)	13,183 (16.5)	6,840 (16.7)	**<0.001**
High blood pressure	133,108 (54.0)	103,722 (54.5)	11,893 (55.0)	17,493 (50.7)	**<0.001**	66,601 (52.9)	43,148 (54.1)	23,359 (57.0)	**<0.001**
Diabetes	56,033 (22.7)	42,560 (22.4)	5,144 (23.8)	8,329 (24.1)	**<0.001**	27,067 (21.5)	18,152 (22.8)	10,814 (26.4)	**<0.001**
Alzheimer's disease/dementia	32,059 (13.0)	24,476 (12.9)	2,776 (12.8)	4,807 (15.0)	**<0.001**	16,430 (13.1)	10,300 (12.9)	5,329 (13.0)	0.668
Parkinson's disease	9,921 (4.0)	7,552 (4.0)	892 (4.1)	1,477 (4.3)	0.018	5,020 (4.0)	3,238 (4.1)	1,663 (4.1)	0.673
Severe depression	35,537 (14.4)	27,326 (14.4)	3,343 (15.5)	4,868 (14.1)	**<0.001**	17,011 (13.5)	12,185 (15.3)	6,341 (15.5)	**<0.001**

Supplementary Table S3. Comparison of characteristics of participants who were excluded in the analyses versus included individuals (n = 502,490).

		Overall	Excluded	Included	P value
**N**		502,490	255,995	246,495	
**Age at baseline, (mean [SD])**		56.5 (8.1)	58.1 (7.8)	54.8 (8.1)	<0.001
**Sex, n (%)**	Female	273,375 (54.4)	120,932 (47.2)	152,443 (61.8)	<0.001
	Male	229,114 (45.6)	135,062 (52.8)	94,052 (38.2)	
**Ethnicity, n (%)**	White	472,680 (94.1)	234,101 (91.4)	238,579 (96.8)	<0.001
	Non-white	27,033 (5.4)	19,690 (7.7)	7,343 (3.0)	
	Unknown	2,777 (0.6)	2,204 (0.9)	573 (0.2)	
**Town deprivation index, n (%)**	Low deprivation	167,374 (33.3)	80,713 (31.5)	86,661 (35.2)	<0.001
	Moderate deprivation	167,210 (33.3)	83,142 (32.5)	84,068 (34.1)	
	High deprivation	167,282 (33.3)	91,843 (35.9)	75,439 (30.6)	
	Unknown	624 (0.1)	297 (0.1)	327 (0.1)	
**Education levels, n (%)**	College or above	161,158 (32.1)	75,457 (29.5)	85,701 (34.8)	<0.001
	Below high school	275,878 (54.9)	149,018 (58.2)	126,860 (51.5)	
	Unknown	65,454 (13.0)	31,520 (12.3)	33,934 (13.8)	
**BMI at baseline (%)**	Normal weight	153,375 (30.5)	72,778 (28.4)	80,597 (32.7)	<0.001
	Underweight	11,769 (2.3)	5,349 (2.1)	6,420 (2.6)	
	Overweight	212,092 (42.2)	110,600 (43.2)	101,492 (41.2)	
	Obesity	122,149 (24.3)	64,163 (25.1)	57,986 (23.5)	
	Unknown	3,105 (0.6)	3,105 (1.2)	0	
**Current smoking status, n (%)**	Never	273,514 (54.4)	132,006 (51.6)	141,508 (57.4)	<0.001
	Previous	173,050 (34.4)	91,851 (35.9)	81,199 (32.9)	
	Current	52,977 (10.5)	29,854 (11.7)	23,123 (9.4)	
	Unknown	2,949 (0.6)	2,284 (0.9)	665 (0.3)	
**Current drinking status, n (%)**	Never	22,385 (4.5)	13,808 (5.4)	8,577 (3.5)	<0.001
	Previous	18,102 (3.6)	10,384 (4.1)	7,718 (3.1)	
	Current	460,349 (91.6)	230,310 (90.0)	230,039 (93.3)	
	Unknown	1,654 (0.3)	1,493 (0.6)	161 (0.1)	
**Physical activity, n (%)**	Low	76,215 (15.2)	39,504 (15.4)	36,711 (14.9)	<0.001
	Moderate	164,017 (32.6)	80,551 (31.5)	83,466 (33.9)	
	High	162,136 (32.3)	79,358 (31.0)	82,778 (33.6)	
	Unknown	100,122 (19.9)	56,582 (22.1)	43,540 (17.7)	
**Intake of fruits, n (%)**	< 2.0 servings/day	167,537 (33.3)	89,481 (35.0)	78,056 (31.7)	<0.001
	2.0-2.9 servings/day	126,759 (25.2)	63,121 (24.7)	63,638 (25.8)	
	3.0-3.9 servings/day	96,901 (19.3)	47,360 (18.5)	49,541 (20.1)	
	≥ 4.0 servings/day	111,293 (22.1)	56,033 (21.9)	55,260 (22.4)	
**Intake of vegetables, n (%)**	< 2.0 servings/day	179,044 (35.6)	95,961 (37.5)	83,083 (33.7)	<0.001
	2.0-2.9 servings/day	167,431 (33.3)	82,659 (32.3)	84,772 (34.4)	
	3.0-3.9 servings/day	87,915 (17.5)	42,800 (16.7)	45,115 (18.3)	
	≥ 4.0 servings/day	68,100 (13.6)	34,575 (13.5)	33,525 (13.6)	
**Maternal smoking around birth, n (%)**	No	306,234 (60.9)	148,536 (58.0)	157,698 (64.0)	<0.001
	Yes	126,623 (25.2)	62,410 (24.4)	64,213 (26.1)	
	Unknown	69,633 (13.9)	45,049 (17.6)	24,584 (10.0)	
**Breastfed as a baby, n (%)**	No	106,114 (21.1)	40,060 (15.6)	66,054 (26.8)	<0.001
	Yes	277,591 (55.2)	121,989 (47.7)	155,602 (63.1)	
	Unknown	118,785 (23.6)	93,946 (36.7)	24,839 (10.1)	

Supplementary Table S4. Factor loadings of each chronic condition for the six multimorbidity patterns.

	Vascular-metabolic-cancer Pattern	Cardiovascular Pattern	Mental Pattern	Respiratory Pattern	Digestive Pattern	Skeletal Pattern
Cancer	**0.42**	-	-	-	-	-
Chronic kidney disease	**0.40**	-	-	-	-	-
Diabetes	**0.59**	-	-	-	-	-
Hypertension	**0.61**	-	-	-	-	-
Thyroid problem	**0.39**	-	-	-	-	-
Angina	-	**0.65**	-	-	-	-
Heart failure	-	**0.47**	-	-	-	-
Myocardial infarction	-	**0.71**	-	-	-	-
Anxiety	-	-	**0.72**	-	-	-
Depression	-	-	**0.72**	-	-	-
Schizophrenia	-	-	**0.36**	-	-	-
Asthma	-	-	-	**0.57**	-	-
Bronchiectasis	-	-	-	**0.61**	-	-
Chronic obstructive pulmonary disease	-	-	-	**0.59**	-	-
Chronic sinusitis	-	-	-	**0.39**	-	-
Irritable bowel syndrome	-	-	-	-	**0.55**	-
Dyspepsia	-	-	-	-	**0.50**	-
Constipation	-	-	-	-	**0.38**	-
Diverticular disease of intestine	-	-	-	-	**0.42**	-
Cirrhosis	-	-	-	-	-	-
Osteoporosis	-	-	-	-	-	**0.61**
Rheumatoid arthritis	-	-	-	-	-	**0.63**
Atrial fibrillation	-	-	-	-	-	-
Dementia	-	-	-	-	-	-
Eczema or dermatitis	-	-	-	-	-	-
Epilepsy	-	-	-	-	-	-
Glaucoma	-	-	-	-	-	-
Hepatitis	-	-	-	-	-	-
Inflammatory bowel disease	-	-	-	-	-	-
Migraine	-	-	-	-	-	-
Multiple sclerosis	-	-	-	-	-	-
Parkinson's disease	-	-	-	-	-	-
Prostate problem	-	-	-	-	-	-
Peripheral vascular disease	-	-	-	-	-	-
Stroke	-	-	-	-	-	-
Hearing loss	-	-	-	-	-	-
Endometriosis	-	-	-	-	-	-
Meniere's disease	-	-	-	-	-	-

Supplementary Table S5. Estimated ORs (95% CIs) for the associations of birth weight and childhood body size with multimorbidity patterns.

**Birth weight**	**Low birth weight**		**High birth weight**	
**Multimorbidity Patterns**	**n (%)**	**OR (95% CI)**	**n (%)**	**OR (95% CI)**
Vascular-metabolic-cancer Pattern	1605 (7.4)	1.58 (1.49-1.67)	1803 (5.2)	1.02 (0.97-1.07)
Cardiovascular Pattern	1426 (6.6)	1.41 (1.33-1.50)	1995 (5.8)	1.18 (1.12-1.24)
Mental Pattern	1313 (6.1)	1.25 (1.18-1.33)	1667 (4.8)	0.97 (0.92-1.03)
Respiratory Pattern	1199 (5.5)	1.54 (1.44-1.64)	1409 (4.1)	1.08 (1.02-1.14)
Digestive Pattern	1446 (6.7)	1.24 (1.17-1.31)	1905 (5.5)	1.02 (0.97-1.07)
Skeletal Pattern	1363 (6.3)	1.34 (1.26-1.42)	1788 (5.2)	1.09 (1.04-1.15)
**Childhood body size**	**Thinner**		**Plumper**	
**Multimorbidity Patterns**	**n (%)**	**OR (95% CI)**	**n (%)**	**OR (95% CI)**
Vascular-metabolic-cancer Pattern	4155 (5.2)	1.10 (1.06-1.14)	2537 (6.2)	1.02 (0.97-1.07)
Cardiovascular Pattern	4236 (5.3)	1.24 (1.19-1.29)	2162 (5.3)	0.94 (0.89-0.99)
Mental Pattern	4185 (5.3)	1.25 (1.20-1.30)	2365 (5.8)	1.17 (1.12-1.23)
Respiratory Pattern	3396 (4.3)	1.32 (1.26-1.38)	1780 (4.3)	1.11 (1.05-1.18)
Digestive Pattern	4875 (6.1)	1.20 (1.16-1.25)	2301 (5.6)	1.06 (1.01-1.11)
Skeletal Pattern	4222 (5.3)	1.10 (1.06-1.15)	2073 (5.1)	1.05 (1.00-1.11)

The ORs and 95% CIs refers to the association of birth weight and childhood body size with the highest group of factor scores of multimorbidity patterns compared with the lowest group.
Adjusted for age at the end of follow-up, sex, ethnicity, Townsend deprivation index, education level, smoking status, drinking status, physical activity, intake of fruits and vegetables, BMI categories at baseline, maternal smoking around birth and breastfed as a baby.

Supplementary Table S6. Sensitivity analysis of the association between birth weight categories and the incidence of multimorbidity.

	Birth weight <2.5 kg, n=21,626 HR (95% CI)	Birth weight 2.5-4.0 kg, n=190,359 HR (95% CI)	Birth weight 4.0-4.5 kg, n=22,064 HR (95% CI)	Birth weight >4.5 kg, n=12,446 HR (95% CI)
**Model 1**	1.32 (1.28-1.35)	Reference	0.98 (0.96-1.01)	1.15 (1.11-1.19)
**Model 2**	1.29 (1.26-1.330	Reference	0.98 (0.95-1.00)	1.10 (1.06-1.14)
**Model 3**	1.29 (1.26-1.33)	Reference	0.98 (0.95-1.00)	1.10 (1.06-1.14)
**Model 4**	1.29 (1.26-1.33)	Reference	0.98 (0.95-1.00)	1.10 (1.06-1.14)

Model 1: unadjusted.
Model 2: adjusted for age at baseline, sex, ethnicity, Townsend deprivation index, education levels, childhood body size and BMI at baseline.
Model 3: model 2 also adjusted for current smoking and drinking status, physical activity, intake of fruits and vegetables.
Model 4: model 3 also adjusted for maternal smoking around birth and breastfed as a baby.

Supplementary Table S7. Sensitivity analysis of the association of birth weight and childhood body size with the incidence of multimorbidity by excluding individuals with missing information on covariates (n=139,546).

**Birth weight**				
**Model**	**Model 1 HR (95% CI)**	**Model 2 HR (95% CI)**	**Model 3 HR (95% CI)**	**Model 4 HR (95% CI)**
Low birth weight, n=12,354	1.34 (1.29-1.39)	1.30 (1.25-1.34)	1.30 (1.26-1.35)	1.30 (1.26-1.35)
Normal birth weight, n=107,735	Reference	Reference	Reference	Reference
High birth weight, n=19,457	1.03 (1.00-1.07)	1.01 (10.98-1.05)	1.01 (0.98-1.05)	1.01 (0.98-1.05)
**Childhood body size**				
**Model**	**Model 1 HR (95% CI)**	**Model 2 HR (95% CI)**	**Model 3 HR (95% CI)**	**Model 4 HR (95% CI)**
Thinner, n=45,249	1.22 (1.19-1.25)	1.20 (1.17-1.23)	1.20 (1.17-1.23)	1.20 (1.17-1.23)
Average, n=70,958	Reference	Reference	Reference	Reference
Plumper, n=13,339	1.05 (1.02-1.08)	1.07 (1.03-1.10)	1.07 (1.03-1.10)	1.07 (1.03-1.10)

Model 1: unadjusted.
Model 2: adjusted for age at baseline, sex, ethnicity, Townsend deprivation index, education levels, and BMI at baseline.
Model 3: model 2 also adjusted for current smoking and drinking status, physical activity, intake of fruits and vegetables.
Model 4: model 3 also adjusted for maternal smoking around birth and breastfed as a baby.
We adjusted for the other variable in each of the birth weight and childhood obesity status models.

Supplementary Table S8. Sensitivity analysis of the association of birth weight and childhood body size with the incidence of multimorbidity by imputing missing covariates with multiple imputation.

**Birth weight**				
**Model**	**Model 1 HR (95% CI)**	**Model 2 HR (95% CI)**	**Model 3 HR (95% CI)**	**Model 4 HR (95% CI)**
Low birth weight, n=21,626	1.29 (1.26-1.33)	1.29 (1.26-1.33)	1.29 (1.26-1.33)	1.29 (1.26-1.33)
Normal birth weight, n=190,359	Reference	Reference	Reference	Reference
High birth weight, n=34,510	1.02 (1.00-1.05)	1.02 (1.00-1.05)	1.02 (1.00-1.05)	1.02 (1.00-1.05)
**Childhood body size**				
**Model**	**Model 1 HR (95% CI)**	**Model 2 HR (95% CI)**	**Model 3 HR (95% CI)**	**Model 4 HR (95% CI)**
Thinner, n=79,700	1.20 (1.18-1.23)	1.20 (1.18-1.23)	1.20 (1.18-1.23)	1.20 (1.18-1.23)
Average, n=125,803	Reference	Reference	Reference	Reference
Plumper, n=40,992	1.06 (1.04-1.09)	1.06 (1.04-1.09)	1.06 (1.04-1.09)	1.06 (1.04-1.09)

Model 1: unadjusted.
Model 2: adjusted for age at baseline, sex, ethnicity, Townsend deprivation index, education levels, and BMI at baseline.
Model 3: model 2 also adjusted for current smoking and drinking status, physical activity, intake of fruits and vegetables.
Model 4: model 3 also adjusted for maternal smoking around birth and breastfed as a baby.
We adjusted for the other variable in each of the birth weight and childhood obesity status models.

Supplementary Table S9. Sensitivity analysis of the association of birth weight, childhood body size, and their changes with the incidence of multimorbidity by additionally adjusting family history of major chronic diseases.

Categories of birth weight, childhood body size, and weight change	HR (95% CI)
**Birth weight**	
Low birth weight	1.26 (1.22-1.29)
Normal birth weight	Reference
High birth weight	1.02 (>1.00-1.04)
**Childhood body size**	
Thinner body size	1.19 (1.17-1.21)
Average body size	Reference
Plumper body size	1.04 (1.02-1.07)
**Weight change**	
Low birth weight→Thinner	1.19 (1.13-1.26)
Low birth weight→Average	Reference
Low birth weight→Plumper	1.12 (1.04-1.21)
Normal birth weight→Thinner	1.19 (1.16-1.21)
Normal birth weight→Average	Reference
Normal birth weight→Plumper	1.03 (1.01-1.06)
High birth weight→Thinner	1.20 (1.14-1.26)
High birth weight→Average	Reference
High birth weight→Plumper	1.06 (1.01-1.12)

All models were adjusted for age at baseline, sex, ethnicity, Townsend deprivation index, education levels, BMI at baseline, current smoking and drinking status, physical activity, intake of fruits and vegetables, maternal smoking around birth, breastfed as a baby, and family history of heart disease, stroke, cancer, chronic bronchitis/emphysema, high blood pression, diabetes, Alzheimer's disease/dementia, Parkinson's disease, and severe depression.

We adjusted for the other variable in each of the birth weight and childhood obesity status models.

Supplementary Figures

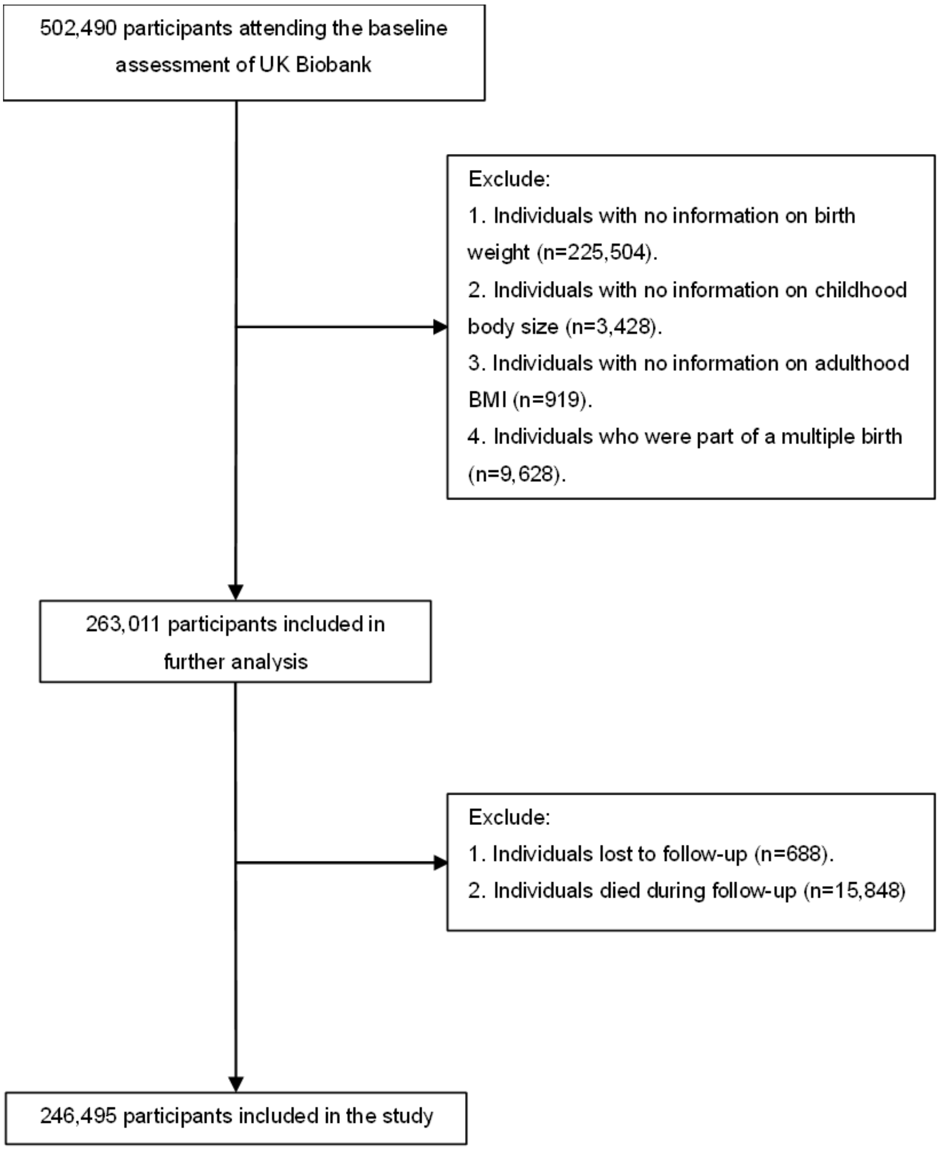

Supplementary Figure S1. Flow chart of the selection process.

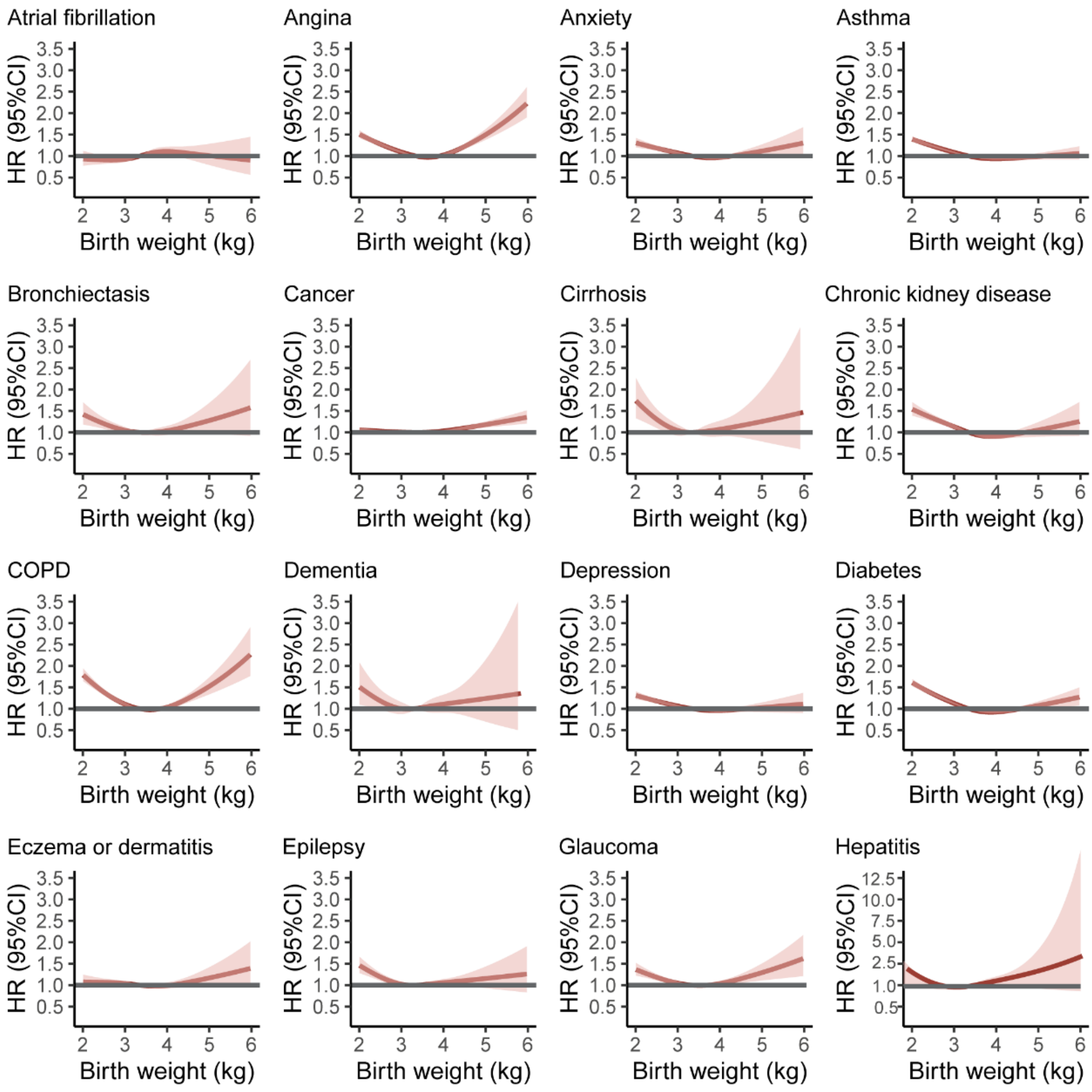

Supplementary Figure S2. Restricted cubic spline models for the relationship of birth weight with the incidence of part of individual chronic conditions.

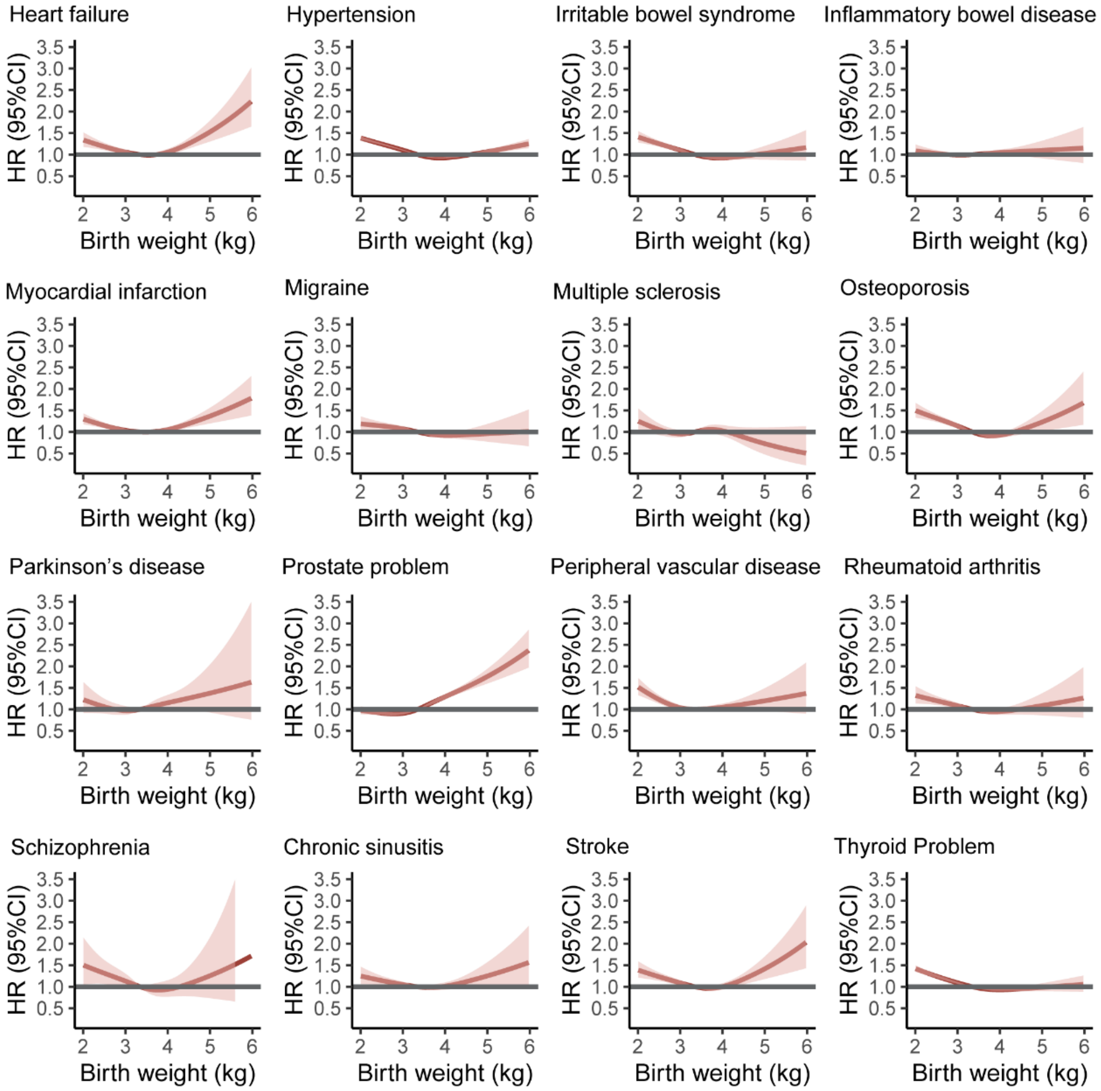

Supplementary Figure S3. Restricted cubic spline models for the relationship of birth weight with the incidence of part of individual chronic conditions.

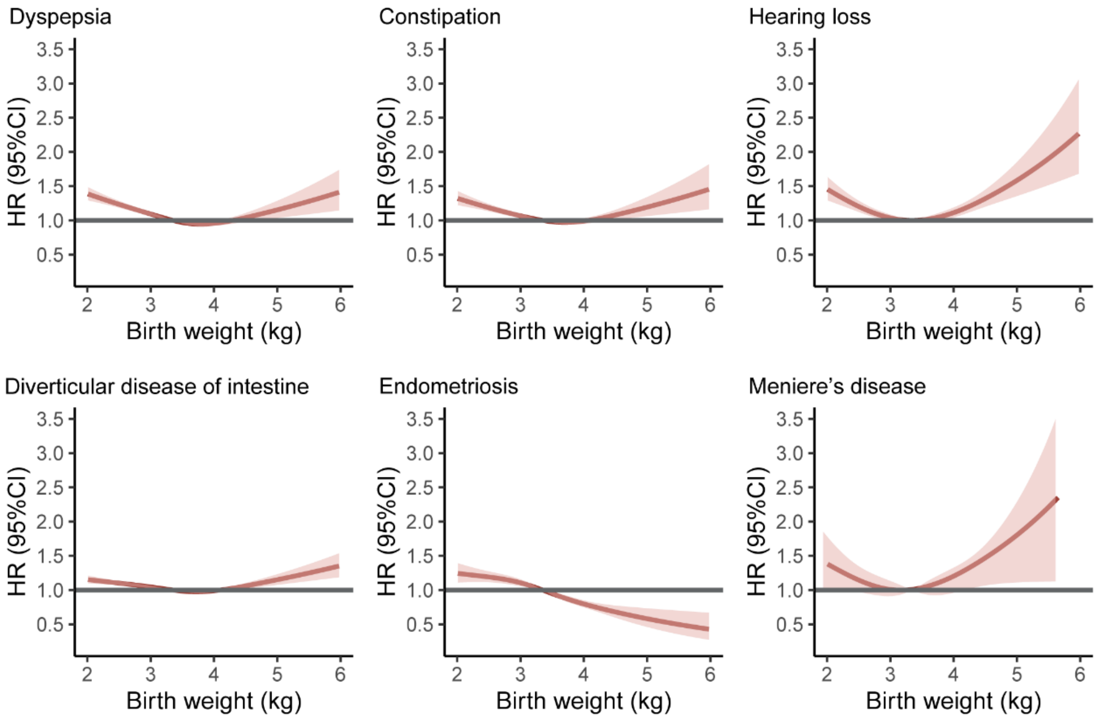

Supplementary Figure S4. Restricted cubic spline models for the relationship of birth weight with the incidence of the remaining individual chronic conditions.

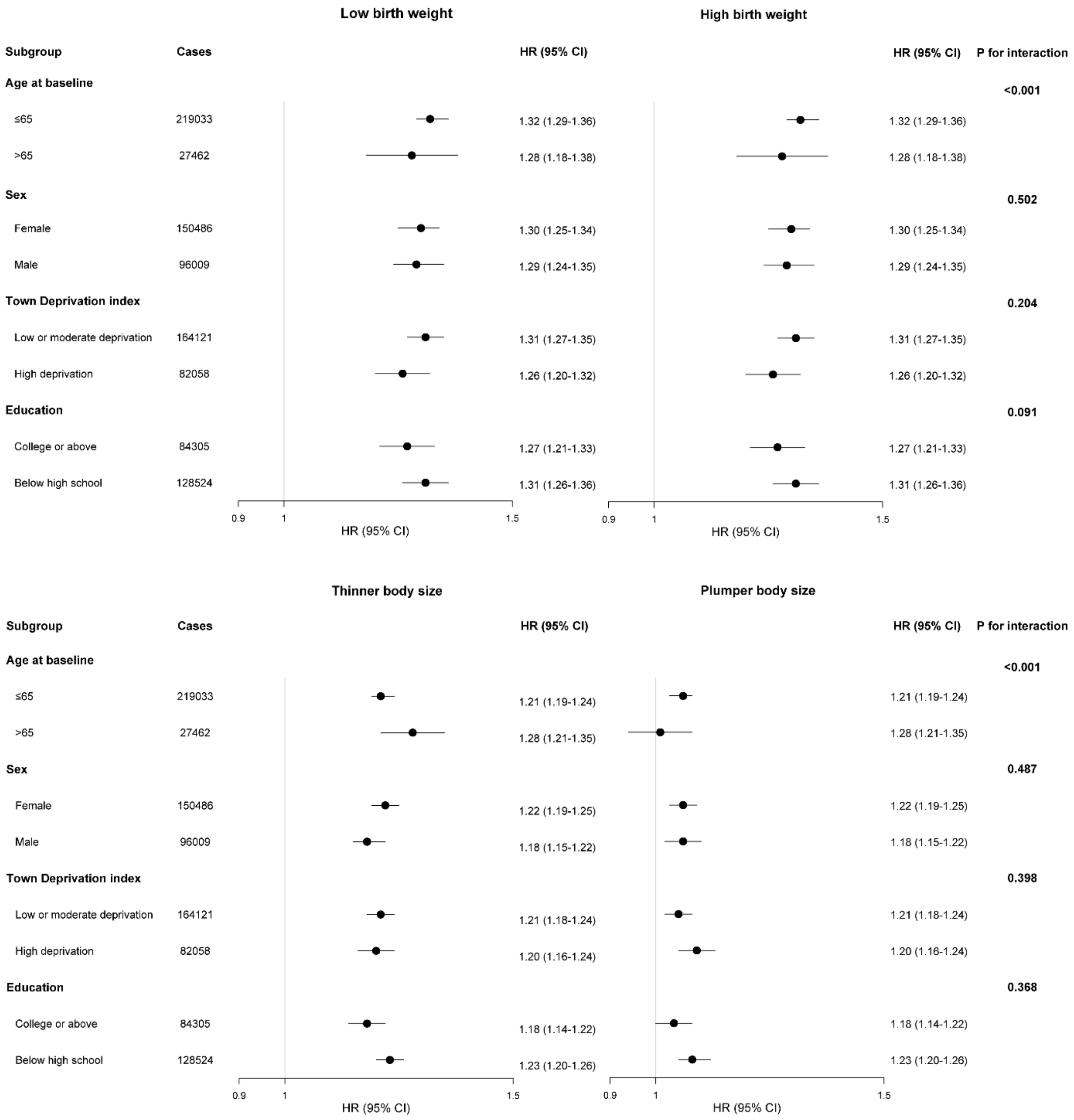

Supplementary Figure S5. Subgroup analysis of the association of birth weight and childhood body size with the incidence of multimorbidity.

The error bars refer to the 95% confidence interval.

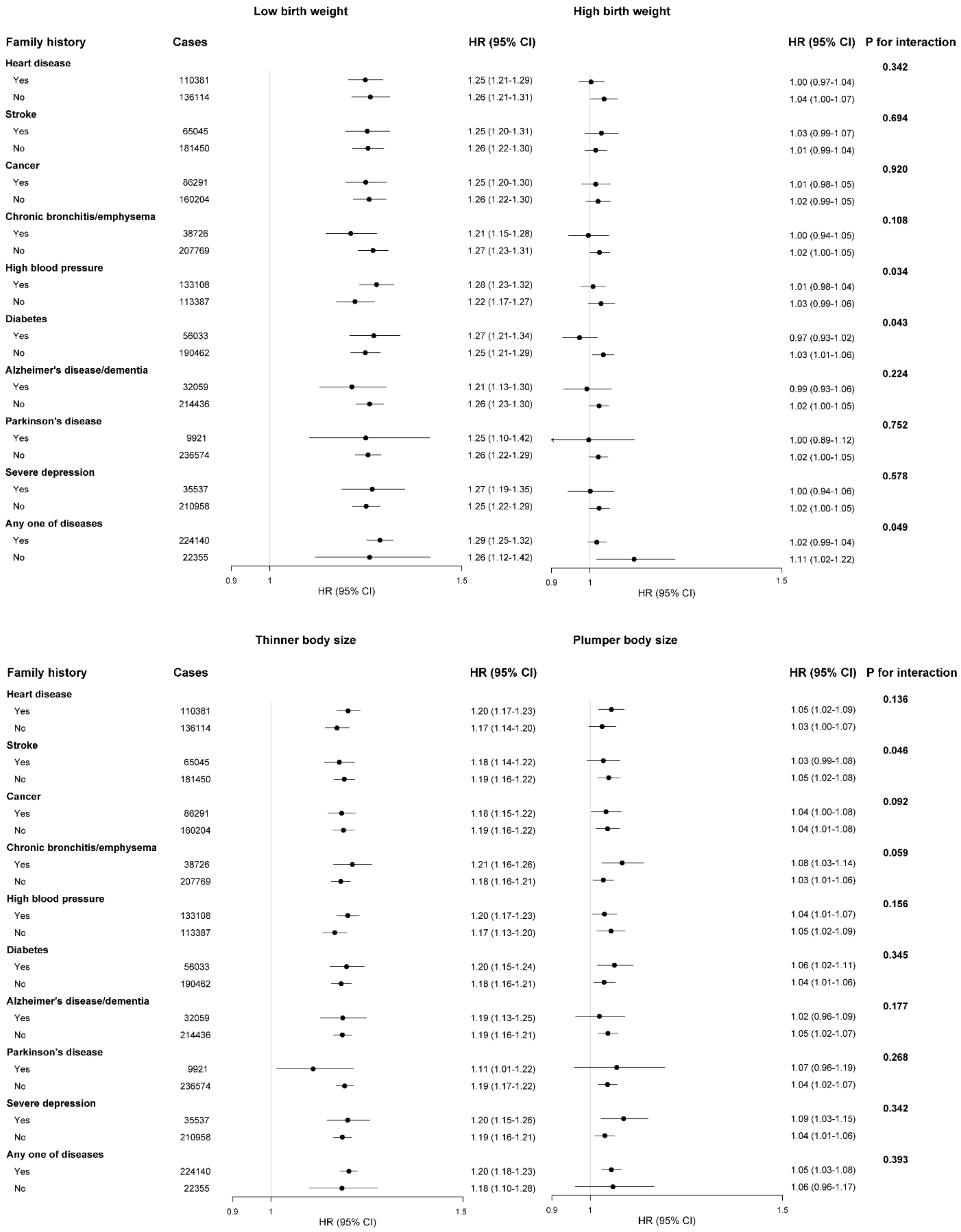

Supplementary Figure S6. Association of birth weight and childhood body size with the incidence of multimorbidity stratified by family history of major disease.
The error bars refer to the 95% confidence interval.
