# Supplementary material for: Association of birth and childhood weight with risk of chronic diseases and multimorbidity in adulthood
Source: Commun Med (Lond). 2023 Jul 31;3:105. doi: 10.1038/s43856-023-00335-4 (PMC10390459; doi:10.1038/s43856-023-00335-4)
Supplement: Supplementary file 3 — Description of Additional Supplementary Data [file 43856_2023_335_MOESM3_ESM.docx]

**Description of Additional Supplementary Files**

**File name:** Supplementary Data 1

**Description:** Basic characteristics of participants by birth weight and childhood body size.

**File name:** Supplementary Data 2

**Description:** Basic characteristics of participants by multimorbidity status during follow-up.
