# Supplementary material for: Association of birth and childhood weight with risk of chronic diseases and multimorbidity in adulthood
Source: Commun Med (Lond). 2023 Jul 31;3:105. doi: 10.1038/s43856-023-00335-4 (PMC10390459; doi:10.1038/s43856-023-00335-4)
Supplement: Supplementary file 6 — Supplementary Data 3 [file 43856_2023_335_MOESM6_ESM.docx]

Main analysis code was provided. Date dictionary could be found in the UK Biobank website (https://www.ukbiobank.ac.uk/.). The meaning of main variables used in the analysis code are shown in the table below.

| **Variables used in the code** | **Meaning** |
| --- | --- |
| bw_c | Birth weight |
| child_obe | Childhood body size |
| mmage | Age at multimorbidity |
| mm | Had multimorbidity |
| bmic | Classification of body mass index |
| ethnic | Ethnicity |
| tdic | Classification of Town Deprivation Index |
| edu | Education level |
| smoke | Current smoking status |
| drink | Current drinking status |
| act | Physical activity |
| fafruit | Intake of fruits |
| faveg | Intake of vegetables |
| m_smoke | Maternal smoking around birth and |
| breast | Breastfed as a baby |

**Code for Table 1**

/*Association between birthweight, childhood obesity, adulthood obesity with the incidence of multimorbidity*/

/*Model 1*/

**proc** **phreg** data=all;

class bw_c(ref='0') child_obe(ref='0') bmic(ref='0');

model mmage*mm(**0**)=bw_c child_obe /risklimits;

**run**;

/*Model 2*/

**proc** **phreg** data=all;

class bw_c(ref='0') child_obe(ref='0') bmic(ref='0') ethnic(ref='0') tdic(ref='0') edu(ref='0');

model mmage*mm(**0**)=bw_c child_obe bmic sex age_base ethnic tdic edu/risklimits;

**run**;

/*Model 3*/

**proc** **phreg** data=all;

class bw_c(ref='0') child_obe(ref='0') bmic(ref='0') ethnic(ref='0') tdic(ref='0') edu(ref='0') smoke(ref='0') drink(ref='0') act(ref='0') fafruit(ref='0') faveg(ref='0');

model mmage*mm(**0**)=bw_c child_obe bmic sex age_base ethnic tdic edu

smoke drink act fafruit faveg/risklimits;

**run**;

/*Model 4*/

**proc** **phreg** data=all;

class bw_c(ref='0') child_obe(ref='0') bmic(ref='0') ethnic(ref='0') tdic(ref='0') edu(ref='0') smoke(ref='0') drink(ref='0') act(ref='0') fafruit(ref='0') faveg(ref='0') m_smoke(ref='0') breast(ref='0');

model mmage*mm(**0**)=bw_c child_obe bmic sex age_base ethnic tdic edu

smoke drink act fafruit faveg m_smoke breast /risklimits;

**run**;

/*Association of body size changes from birth to childhood (aged 10)

with the incidence of multimorbidity.*/

**data** all;

set all;

if bw_c=**0** and child_obe=**0** then change=**0**; *normal to normal;

else if bw_c=**1** and child_obe=**1** then change=**1**; *low birth weight to thinner;

else if bw_c=**1** and child_obe=**0** then change=**2**; *low birth weight to normal;

else if bw_c=**1** and child_obe=**2** then change=**3**; *low birth weight to plumper;

else if bw_c=**0** and child_obe=**1** then change=**4**; *normal to thinner;

else if bw_c=**0** and child_obe=**2** then change=**5**; *normal to plumper;

else if bw_c=**2** and child_obe=**1** then change=**6**; *high birth weight to thinner;

else if bw_c=**2** and child_obe=**0** then change=**7**; *high birth weight to normal;

else if bw_c=**2** and child_obe=**2** then change=**8**; *high birth weight to plumper;

**run**;

**data** low normal high;

set all;

if bw_c=**0** then output normal;

else if bw_c=**1** then output low;

else output high;

**run**;

/*Low birth weight */

/*Model 1*/

**proc** **phreg** data=low;

class child_obe(ref='0') bmic(ref='0');

model mmage*mm(**0**)=child_obe/risklimits;

**run**;

/*Model 2*/

**proc** **phreg** data=low;

class child_obe(ref='0') bmic(ref='0') ethnic(ref='0') tdic(ref='0') edu(ref='0');

model mmage*mm(**0**)=child_obe bmic sex age_base ethnic tdic edu/risklimits;

**run**;

/*Model 3*/

**proc** **phreg** data=low;

class child_obe(ref='0') bmic(ref='0') ethnic(ref='0') tdic(ref='0') edu(ref='0')

smoke(ref='0') drink(ref='0') act(ref='0') fafruit(ref='0') faveg(ref='0');

model mmage*mm(**0**)=child_obe bmic sex age_base ethnic tdic edu

smoke drink act fafruit faveg/risklimits;

**run**;

/*Model 4*/

**proc** **phreg** data=low;

class child_obe(ref='0') bmic(ref='0') ethnic(ref='0') tdic(ref='0') edu(ref='0')

smoke(ref='0') drink(ref='0') act(ref='0') fafruit(ref='0') faveg(ref='0') m_smoke(ref='0') breast(ref='0');

model mmage*mm(**0**)=child_obe bmic sex age_base ethnic tdic edu

smoke drink act fafruit faveg m_smoke breast/risklimits;

**run**;

/*Normal birth weight */

/*Model 1*/

**proc** **phreg** data=normal;

class child_obe(ref='0') bmic(ref='0');

model mmage*mm(**0**)=child_obe/risklimits;

**run**;

/*Model 2*/

**proc** **phreg** data=normal;

class child_obe(ref='0') bmic(ref='0') ethnic(ref='0') tdic(ref='0') edu(ref='0');

model mmage*mm(**0**)=child_obe bmic sex age_base ethnic tdic edu/risklimits;

**run**;

/*Model 3*/

**proc** **phreg** data=normal;

class child_obe(ref='0') bmic(ref='0') ethnic(ref='0') tdic(ref='0') edu(ref='0')

smoke(ref='0') drink(ref='0') act(ref='0') fafruit(ref='0') faveg(ref='0');

model mmage*mm(**0**)=child_obe bmic sex age_base ethnic tdic edu

smoke drink act fafruit faveg/risklimits;

**run**;

/*Model 4*/

**proc** **phreg** data=normal;

class child_obe(ref='0') bmic(ref='0') ethnic(ref='0') tdic(ref='0') edu(ref='0')

smoke(ref='0') drink(ref='0') act(ref='0') fafruit(ref='0') faveg(ref='0') m_smoke(ref='0') breast(ref='0');

model mmage*mm(**0**)=child_obe bmic sex age_base ethnic tdic edu

smoke drink act fafruit faveg m_smoke breast /risklimits;

**run**;

/*High birth weight */

/*Model 1*/

**proc** **phreg** data=high;

class child_obe(ref='0') bmic(ref='0');

model mmage*mm(**0**)=child_obe/risklimits;

**run**;

/*Model 2*/

**proc** **phreg** data=high;

class child_obe(ref='0') bmic(ref='0') ethnic(ref='0') tdic(ref='0') edu(ref='0');

model mmage*mm(**0**)=child_obe bmic sex age_base ethnic tdic edu/risklimits;

**run**;

/*Model 3*/

**proc** **phreg** data=high;

class child_obe(ref='0') bmic(ref='0') ethnic(ref='0') tdic(ref='0') edu(ref='0')

smoke(ref='0') drink(ref='0') act(ref='0') fafruit(ref='0') faveg(ref='0');

model mmage*mm(**0**)=child_obe bmic sex age_base ethnic tdic edu

smoke drink act fafruit faveg/risklimits;

**run**;

/*Model 4*/

**proc** **phreg** data=high;

class child_obe(ref='0') bmic(ref='0') ethnic(ref='0') tdic(ref='0') edu(ref='0')

smoke(ref='0') drink(ref='0') act(ref='0') fafruit(ref='0') faveg(ref='0') m_smoke(ref='0') breast(ref='0');

model mmage*mm(**0**)=child_obe bmic sex age_base ethnic tdic edu

smoke drink act fafruit faveg m_smoke breast /risklimits;

**run**;

**Code for Table 2**

/*Association of birth weight and childhood body size with number of chronic conditions*/

/*model 1*/

**proc** **glimmix** data = all;

class bw_c(ref='0') child_obe(ref='0');

model num_mm = bw_c child_obe /link = log cl solution;

_variance_ = _mu;

random _residual;

estimate 'bw_c10' bw_c **1** **0** -**1**/ exp cl;

estimate 'bw_c20' bw_c **0** **1** -**1**/ exp cl;

estimate 'child_obe10' child_obe **1** **0** -**1**/ exp cl;

estimate 'child_obec20' child_obe **0** **1** -**1**/ exp cl;

**run**;

/*model 2*/

**proc** **glimmix** data = all;

class bw_c(ref='0') child_obe(ref='0') bmic(ref='0') ethnic(ref='0') tdic(ref='0') edu(ref='0');

model num_mm = bw_c child_obe bmic sex age_base ethnic tdic edu/link = log cl solution;

_variance_ = _mu;

random _residual;

estimate 'bw_c10' bw_c **1** **0** -**1**/ exp cl;

estimate 'bw_c20' bw_c **0** **1** -**1**/ exp cl;

estimate 'child_obe10' child_obe **1** **0** -**1**/ exp cl;

estimate 'child_obec20' child_obe **0** **1** -**1**/ exp cl;

**run**;

/*model 3*/

**proc** **glimmix** data = all;

class bw_c(ref='0') child_obe(ref='0') bmic(ref='0') ethnic(ref='0') tdic(ref='0') edu(ref='0')

smoke(ref='0') drink(ref='0') act(ref='0') fafruit(ref='0') faveg(ref='0');

model num_mm = bw_c child_obe bmic sex age_base ethnic tdic edu smoke drink act fafruit faveg/link = log cl solution;

_variance_ = _mu;

random _residual;

estimate 'bw_c10' bw_c **1** **0** -**1**/ exp cl;

estimate 'bw_c20' bw_c **0** **1** -**1**/ exp cl;

estimate 'child_obe10' child_obe **1** **0** -**1**/ exp cl;

estimate 'child_obec20' child_obe **0** **1** -**1**/ exp cl;

**run**;

/*model 4*/

**proc** **glimmix** data = all;

class bw_c(ref='0') child_obe(ref='0') bmic(ref='0') ethnic(ref='0') tdic(ref='0') edu(ref='0')

smoke(ref='0') drink(ref='0') act(ref='0') fafruit(ref='0') faveg(ref='0') m_smoke(ref='0') breast(ref='0');

model num_mm = bw_c child_obe bmic sex age_base ethnic tdic edu smoke drink act fafruit faveg m_smoke breast/link = log cl solution;

_variance_ = _mu;

random _residual;

estimate 'bw_c10' bw_c **1** **0** -**1**/ exp cl;

estimate 'bw_c20' bw_c **0** **1** -**1**/ exp cl;

estimate 'child_obe10' child_obe **1** **0** -**1**/ exp cl;

estimate 'child_obec20' child_obe **0** **1** -**1**/ exp cl;

**run**;

/*Association of weight change with number of chronic conditions*/

/*Low birth weight */

**proc** **glimmix** data = low;

class child_obe(ref='0') bmic(ref='0') ethnic(ref='0') tdic(ref='0') edu(ref='0')

smoke(ref='0') drink(ref='0') act(ref='0') fafruit(ref='0') faveg(ref='0') m_smoke(ref='0') breast(ref='0');

model num_mm =child_obe bmic sex age_base ethnic tdic edu

smoke drink act fafruit faveg m_smoke breast/link = log cl solution;

_variance_ = _mu;

random _residual;

estimate 'child_obe10' child_obe **1** **0** -**1**/ exp cl;

estimate 'child_obec20' child_obe **0** **1** -**1**/ exp cl;

**run**;

/*Normal birth weight */

**proc** **glimmix** data = normal;

class child_obe(ref='0') bmic(ref='0') ethnic(ref='0') tdic(ref='0') edu(ref='0')

smoke(ref='0') drink(ref='0') act(ref='0') fafruit(ref='0') faveg(ref='0') m_smoke(ref='0') breast(ref='0');

model num_mm =child_obe bmic sex age_base ethnic tdic edu

smoke drink act fafruit faveg m_smoke breast /link = log cl solution;

_variance_ = _mu;

random _residual;

estimate 'child_obe10' child_obe **1** **0** -**1**/ exp cl;

estimate 'child_obec20' child_obe **0** **1** -**1**/ exp cl;

**run**;

/*High birth weight */

**proc** **glimmix** data = high;

class child_obe(ref='0') bmic(ref='0') ethnic(ref='0') tdic(ref='0') edu(ref='0')

smoke(ref='0') drink(ref='0') act(ref='0') fafruit(ref='0') faveg(ref='0') m_smoke(ref='0') breast(ref='0');

model num_mm =child_obe bmic sex age_base ethnic tdic edu

smoke drink act fafruit faveg m_smoke breast

fheart fstroke fcancer fbronch fhyp fdm fdemen fpark fdep/link = log cl solution;

_variance_ = _mu;

random _residual;

estimate 'child_obe10' child_obe **1** **0** -**1**/ exp cl;

estimate 'child_obec20' child_obe **0** **1** -**1**/ exp cl;

**run**;

**Code for Figure 1** (using R)

##CIF

dd <- all[, c('mmage', 'mm', 'bw_c', 'child_obe', 'bmic', 'bw_l', 'bmi',

'sex', 'age_base', 'tdic', 'edu',

'smoke', 'drink', 'act', 'fafruit', 'faveg', 'm_smoke', 'breast')]

df <- datadist(dd)

options(datadist = 'df')

#birth weight

tiff("D:\\childhood_obesity\\results\\CIF\\CIF_bw.tiff", height = 10, width = 10, units = 'in', res = 600)

p1 <- ggsurvplot(survfit(Surv(mmage, mm) ~ bw_c, data = dd),

pval = TRUE, fun = "event",

size = 1,

linetype = "strata",

break.time.by = 10,

palette = c("#45B39D", "#CD6155","#5499C7"),

# conf.int = TRUE,

title = "Birth weight",

legend = c(0.8, 0.2),

legend.title = "Birth weight",

legend.labs = c("Normal birth weight", "Low birth weight", "High birth weight"),

font.legend = c(14, "black"),

font.title = c(20, "bold", "black"),

font.x = c(18, "bold", "black"),

font.y = c(18, "bold", "black"),

font.tickslab = c(14, "plain", "black"),

xlab = "Age at incident multimorbidity",

ylab = "Cumulative event rate (%)",

xlim = c(40,80), ylim = c(0,0.5),

ggtheme = theme_bw())

dev.off()

p1

#childhood obesity

tiff("D:\\childhood_obesity\\results\\CIF\\CIF_child_obe.tiff", height = 10, width = 10, units = 'in', res = 600)

p2 <- ggsurvplot(survfit(Surv(mmage, mm) ~ child_obe, data = dd),

pval = TRUE, fun = "event",

size = 1,

linetype = "strata",

break.time.by = 10,

palette = c("#45B39D", "#CD6155","#5499C7"),

# conf.int = TRUE,

title = "Childhood body size",

legend = c(0.8,0.2),

legend.title = "Childhood body size",

legend.labs = c("Average", "Thinner", "Plumper"),

font.legend = c(14, "black"),

font.title = c(20, "bold", "black"),

font.x = c(18, "bold", "black"),

font.y = c(18, "bold", "black"),

font.tickslab = c(14, "plain", "black"),

xlab = "Age at incident multimorbidity",

ylab = "Cumulative event rate (%)",

xlim = c(40,80), ylim = c(0,0.5),

ggtheme = theme_bw())

p2

dev.off()

**Code for Figure 2** (using R)

#Restricted cubic spline

dd <- all[, c('mmage', 'mm', 'bw_c', 'child_obe', 'bmic', 'bw_l', 'bmi',

'sex', 'age_base', 'tdic', 'edu',

'smoke', 'drink', 'act', 'fafruit', 'faveg', 'm_smoke', 'breast')]

df <- datadist(dd)

options(datadist = 'df')

##birth weight

fit1 <- cph(Surv(mmage, mm) ~ rcs(bw_l, 4) + child_obe + bmic + sex + age_base +

tdic + edu + smoke + drink + act + fafruit + faveg + m_smoke + breast, data = dd)

cox.zph(fit1, "rank")

ggcoxzph(cox.zph(fit1, "rank"))

HR1 <- Predict(fit1, bw_l, fun=exp, ref.zero = TRUE)

HR1 <- as.data.frame(HR1)

p1 <- ggplot(HR1) +geom_line(aes(bw_l,yhat),linetype=1,size=1,alpha = 0.9,colour='#922B21')+

geom_ribbon(data=HR1, aes(bw_l,ymin = lower, ymax = upper),alpha = 0.5,fill='#E6B0AA')+

geom_hline(yintercept=1, linetype=1,size=1, colour = '#626567')+theme_classic() +

geom_vline(xintercept=3.33, linetype=2,size=0.5)+theme_classic() +

geom_vline(xintercept=4.09, linetype=2,size=0.5)+theme_classic() +

scale_y_continuous(breaks = seq(0.80,2.2,0.2), limits = c(0.8,2.2)) +

scale_x_continuous(breaks = seq(0,7,1), limits = c(0,7)) +

labs(title = "Birth weight", x="Birth weight (kg)", y="HR (95%CI)")

anova(fit1)

**Code for Figure 3**

/*Association of birth weight and childhood body size with incident individual chronic conditions*/

**data** all;

set all;

array disease {**38**} af angina anxiety asthma bronch cancer cirrhosis ckb copd dementia depression dm ed epilepsy glaucoma hepatitis hf hypertension ibs idb mi migraine ms ost parkinson prostate pvd ra sch sinusitis stroke thy dyspepsia constipation hear diverticular endometriosis meniere;

array diseaseage {**38**} fage anginaage anxietyage asthmaage bronchage cancerage cirrhosisage ckbage copdage dementiaage

depressionage dmage edage epilepsyage glaucomaage hepatitisage hfage hypertensionage ibsage idbage miage migraineage msage ostage parkinsonage prostateage pvdage raage schage sinusitisage strokeage thyage dyspepsiaage constipationage hearage diverticularage endometriosisage meniereage;

do i = **1** to **38**;

if disease {i} = **1** then diseaseage {i} = diseaseage {i};

else diseaseage {i} = (**'31DEC2020'd**-s_53_0_0)/**365**+n_21022_0_0;

end;

drop i;

**run**;

libname out xlsx "D:\childhood_obesity\results\HR_for_individual\HR1.xlsx";

**%macro** HR(y= ,age=);

%let outputName=_PE%substr(%cmpres(&y.),1,%sysfunc(min(%length(&y.),29)));

ods graphics off;

ods output ParameterEstimates=&outputName.;

proc phreg data = all;

class bw_c(ref='0') child_obe(ref='0') bmic(ref='0') ethnic(ref='0') tdic(ref='0') edu(ref='0')

smoke(ref='0') drink(ref='0') act(ref='0') fafruit(ref='0') faveg(ref='0') m_smoke(ref='0') breast(ref='0');

model &age*&y(**0**) = bw_c child_obe bmic sex age_base ethnic tdic edu

smoke drink act fafruit faveg m_smoke breast/rl;

run;

ods output close;

ods graphics on;

data out.&outputName.;

set &outputName.;

HazardRatio=round(HazardRatio,**0.01**);

HRLowerCL=round(HRLowerCL,**0.01**);

HRUpperCL=round(HRUpperCL,**0.01**);

format HazardRatio **4.2** HRLowerCL **4.2** HRUpperCL **4.2**;

hr=put(HazardRatio,**4.2**);

lci=put(HRLowerCL,**4.2**);

hci=put(HRUpperCL,**4.2**);

combine=compress(hr)||" ("||compress(lci)||"-"||compress(hci)||")";

keep Parameter ClassVal0 HazardRatio HRLowerCL HRUpperCL hr lci hci combine;

if Parameter="bw_c" or Parameter="child_obe";

run;

**%mend** HR;

%***HR***(y=af, age=afage);

%***HR***(y=angina, age=anginaage);

%***HR***(y=anxiety, age=anxietyage);

%***HR***(y=asthma, age=asthmaage);

%***HR***(y=bronch, age=bronchage);

%***HR***(y=cancer, age=cancerage);

%***HR***(y=cirrhosis, age=cirrhosisage);

%***HR***(y=ckb, age=ckbage);

%***HR***(y=copd, age=copdage);

%***HR***(y=dementia, age=dementiaage);

%***HR***(y=depression, age=depressionage);

%***HR***(y=dm, age=dmage);

%***HR***(y=ed, age=edage);

%***HR***(y=epilepsy, age=epilepsyage);

%***HR***(y=glaucoma, age=glaucomaage);

%***HR***(y=hepatitis, age=hepatitisage);

%***HR***(y=hf, age=hfage);

%***HR***(y=hypertension, age=hypertensionage);

%***HR***(y=ibs, age=ibsage);

%***HR***(y=idb, age=idbage);

%***HR***(y=mi, age=miage);

%***HR***(y=migraine, age=migraineage);

%***HR***(y=ms, age=msage);

%***HR***(y=ost, age=ostage);

%***HR***(y=parkinson, age=parkinsonage);

%***HR***(y=prostate, age=prostateage);

%***HR***(y=pvd, age=pvdage);

%***HR***(y=ra, age=raage);

%***HR***(y=sch, age=schage);

%***HR***(y=sinusitis, age=sinusitisage);

%***HR***(y=stroke, age=strokeage);

%***HR***(y=thy, age=thyage);

%***HR***(y=dyspepsia, age=dyspepsiaage);

%***HR***(y=constipation, age=constipationage);

%***HR***(y=hear, age=hearage);

%***HR***(y=diverticular, age=diverticularage);

%***HR***(y=endometriosis, age=endometriosisage);

%***HR***(y=meniere, age=meniereage);

/*Association of weight change with individual chronic conditions*/

/*Low birth weight */

libname out xlsx "E:\onedrive\childhood_obesity\results\weightchange_indi\low.xlsx";

**%macro** HR(y= ,age=);

%let outputName=_PE%substr(%cmpres(&y.),1,%sysfunc(min(%length(&y.),29)));

ods graphics off;

ods output ParameterEstimates=&outputName.;

proc phreg data = low;

class child_obe(ref='0') bmic(ref='0') ethnic(ref='0') tdic(ref='0') edu(ref='0')

smoke(ref='0') drink(ref='0') act(ref='0') fafruit(ref='0') faveg(ref='0') m_smoke(ref='0') breast(ref='0');

model &age*&y(**0**) = child_obe bmic sex age_base ethnic tdic edu

smoke drink act fafruit faveg m_smoke breast/rl;

run;

ods output close;

ods graphics on;

data out.&outputName.;

set &outputName.;

HazardRatio=round(HazardRatio,**0.01**);

HRLowerCL=round(HRLowerCL,**0.01**);

HRUpperCL=round(HRUpperCL,**0.01**);

format HazardRatio **4.2** HRLowerCL **4.2** HRUpperCL **4.2**;

hr=put(HazardRatio,**4.2**);

lci=put(HRLowerCL,**4.2**);

hci=put(HRUpperCL,**4.2**);

combine=compress(hr)||" ("||compress(lci)||"-"||compress(hci)||")";

keep Parameter ClassVal0 HazardRatio HRLowerCL HRUpperCL hr lci hci combine;

if Parameter="bw_c" or Parameter="child_obe";

run;

**%mend** HR;

%***HR***(y=af, age=afage);

%***HR***(y=angina, age=anginaage);

%***HR***(y=anxiety, age=anxietyage);

%***HR***(y=asthma, age=asthmaage);

%***HR***(y=bronch, age=bronchage);

%***HR***(y=cancer, age=cancerage);

%***HR***(y=cirrhosis, age=cirrhosisage);

%***HR***(y=ckb, age=ckbage);

%***HR***(y=copd, age=copdage);

%***HR***(y=dementia, age=dementiaage);

%***HR***(y=depression, age=depressionage);

%***HR***(y=dm, age=dmage);

%***HR***(y=ed, age=edage);

%***HR***(y=epilepsy, age=epilepsyage);

%***HR***(y=glaucoma, age=glaucomaage);

%***HR***(y=hepatitis, age=hepatitisage);

%***HR***(y=hf, age=hfage);

%***HR***(y=hypertension, age=hypertensionage);

%***HR***(y=ibs, age=ibsage);

%***HR***(y=idb, age=idbage);

%***HR***(y=mi, age=miage);

%***HR***(y=migraine, age=migraineage);

%***HR***(y=ms, age=msage);

%***HR***(y=ost, age=ostage);

%***HR***(y=parkinson, age=parkinsonage);

%***HR***(y=prostate, age=prostateage);

%***HR***(y=pvd, age=pvdage);

%***HR***(y=ra, age=raage);

%***HR***(y=sch, age=schage);

%***HR***(y=sinusitis, age=sinusitisage);

%***HR***(y=stroke, age=strokeage);

%***HR***(y=thy, age=thyage);

%***HR***(y=dyspepsia, age=dyspepsiaage);

%***HR***(y=constipation, age=constipationage);

%***HR***(y=hear, age=hearage);

%***HR***(y=diverticular, age=diverticularage);

%***HR***(y=endometriosis, age=endometriosisage);

%***HR***(y=meniere, age=meniereage);

/*Normal birth weight */

libname out xlsx "E:\onedrive\childhood_obesity\results\weightchange_indi\normal.xlsx";

**%macro** HR(y= ,age=);

%let outputName=_PE%substr(%cmpres(&y.),1,%sysfunc(min(%length(&y.),29)));

ods graphics off;

ods output ParameterEstimates=&outputName.;

proc phreg data = normal;

class child_obe(ref='0') bmic(ref='0') ethnic(ref='0') tdic(ref='0') edu(ref='0')

smoke(ref='0') drink(ref='0') act(ref='0') fafruit(ref='0') faveg(ref='0') m_smoke(ref='0') breast(ref='0');

model &age*&y(**0**) = child_obe bmic sex age_base ethnic tdic edu

smoke drink act fafruit faveg m_smoke breast/rl;

run;

ods output close;

ods graphics on;

data out.&outputName.;

set &outputName.;

HazardRatio=round(HazardRatio,**0.01**);

HRLowerCL=round(HRLowerCL,**0.01**);

HRUpperCL=round(HRUpperCL,**0.01**);

format HazardRatio **4.2** HRLowerCL **4.2** HRUpperCL **4.2**;

hr=put(HazardRatio,**4.2**);

lci=put(HRLowerCL,**4.2**);

hci=put(HRUpperCL,**4.2**);

combine=compress(hr)||" ("||compress(lci)||"-"||compress(hci)||")";

keep Parameter ClassVal0 HazardRatio HRLowerCL HRUpperCL hr lci hci combine;

if Parameter="bw_c" or Parameter="child_obe";

run;

**%mend** HR;

%***HR***(y=af, age=afage);

%***HR***(y=angina, age=anginaage);

%***HR***(y=anxiety, age=anxietyage);

%***HR***(y=asthma, age=asthmaage);

%***HR***(y=bronch, age=bronchage);

%***HR***(y=cancer, age=cancerage);

%***HR***(y=cirrhosis, age=cirrhosisage);

%***HR***(y=ckb, age=ckbage);

%***HR***(y=copd, age=copdage);

%***HR***(y=dementia, age=dementiaage);

%***HR***(y=depression, age=depressionage);

%***HR***(y=dm, age=dmage);

%***HR***(y=ed, age=edage);

%***HR***(y=epilepsy, age=epilepsyage);

%***HR***(y=glaucoma, age=glaucomaage);

%***HR***(y=hepatitis, age=hepatitisage);

%***HR***(y=hf, age=hfage);

%***HR***(y=hypertension, age=hypertensionage);

%***HR***(y=ibs, age=ibsage);

%***HR***(y=idb, age=idbage);

%***HR***(y=mi, age=miage);

%***HR***(y=migraine, age=migraineage);

%***HR***(y=ms, age=msage);

%***HR***(y=ost, age=ostage);

%***HR***(y=parkinson, age=parkinsonage);

%***HR***(y=prostate, age=prostateage);

%***HR***(y=pvd, age=pvdage);

%***HR***(y=ra, age=raage);

%***HR***(y=sch, age=schage);

%***HR***(y=sinusitis, age=sinusitisage);

%***HR***(y=stroke, age=strokeage);

%***HR***(y=thy, age=thyage);

%***HR***(y=dyspepsia, age=dyspepsiaage);

%***HR***(y=constipation, age=constipationage);

%***HR***(y=hear, age=hearage);

%***HR***(y=diverticular, age=diverticularage);

%***HR***(y=endometriosis, age=endometriosisage);

%***HR***(y=meniere, age=meniereage);

/*High birth weight */

libname out xlsx "E:\onedrive\childhood_obesity\results\weightchange_indi\high.xlsx";

**%macro** HR(y= ,age=);

%let outputName=_PE%substr(%cmpres(&y.),1,%sysfunc(min(%length(&y.),29)));

ods graphics off;

ods output ParameterEstimates=&outputName.;

proc phreg data = high;

class child_obe(ref='0') bmic(ref='0') ethnic(ref='0') tdic(ref='0') edu(ref='0')

smoke(ref='0') drink(ref='0') act(ref='0') fafruit(ref='0') faveg(ref='0') m_smoke(ref='0') breast(ref='0');

model &age*&y(**0**) = child_obe bmic sex age_base ethnic tdic edu

smoke drink act fafruit faveg m_smoke breast/rl;

run;

ods output close;

ods graphics on;

data out.&outputName.;

set &outputName.;

HazardRatio=round(HazardRatio,**0.01**);

HRLowerCL=round(HRLowerCL,**0.01**);

HRUpperCL=round(HRUpperCL,**0.01**);

format HazardRatio **4.2** HRLowerCL **4.2** HRUpperCL **4.2**;

hr=put(HazardRatio,**4.2**);

lci=put(HRLowerCL,**4.2**);

hci=put(HRUpperCL,**4.2**);

combine=compress(hr)||" ("||compress(lci)||"-"||compress(hci)||")";

keep Parameter ClassVal0 HazardRatio HRLowerCL HRUpperCL hr lci hci combine;

if Parameter="bw_c" or Parameter="child_obe";

run;

**%mend** HR;

%***HR***(y=af, age=afage);

%***HR***(y=angina, age=anginaage);

%***HR***(y=anxiety, age=anxietyage);

%***HR***(y=asthma, age=asthmaage);

%***HR***(y=bronch, age=bronchage);

%***HR***(y=cancer, age=cancerage);

%***HR***(y=cirrhosis, age=cirrhosisage);

%***HR***(y=ckb, age=ckbage);

%***HR***(y=copd, age=copdage);

%***HR***(y=dementia, age=dementiaage);

%***HR***(y=depression, age=depressionage);

%***HR***(y=dm, age=dmage);

%***HR***(y=ed, age=edage);

%***HR***(y=epilepsy, age=epilepsyage);

%***HR***(y=glaucoma, age=glaucomaage);

%***HR***(y=hepatitis, age=hepatitisage);

%***HR***(y=hf, age=hfage);

%***HR***(y=hypertension, age=hypertensionage);

%***HR***(y=ibs, age=ibsage);

%***HR***(y=idb, age=idbage);

%***HR***(y=mi, age=miage);

%***HR***(y=migraine, age=migraineage);

%***HR***(y=ms, age=msage);

%***HR***(y=ost, age=ostage);

%***HR***(y=parkinson, age=parkinsonage);

%***HR***(y=prostate, age=prostateage);

%***HR***(y=pvd, age=pvdage);

%***HR***(y=ra, age=raage);

%***HR***(y=sch, age=schage);

%***HR***(y=sinusitis, age=sinusitisage);

%***HR***(y=stroke, age=strokeage);

%***HR***(y=thy, age=thyage);

%***HR***(y=dyspepsia, age=dyspepsiaage);

%***HR***(y=constipation, age=constipationage);

%***HR***(y=hear, age=hearage);

%***HR***(y=diverticular, age=diverticularage);

%***HR***(y=endometriosis, age=endometriosisage);

%***HR***(y=meniere, age=meniereage);

**Code for factor analysis**

/*Multimorbidity patterns identified by exploratory factor analysis*/

%let disease=af angina anxiety asthma bronch cancer cirrhosis ckb copd dementia depression dm ed epilepsy glaucoma hepatitis hf hypertension ibs idb mi migraine ms ost parkinson prostate pvd ra sch sinusitis stroke thy

dyspepsia constipation hear diverticular endometriosis meniere;

**data** factor;

set all;

keep n_eid &disease;

**run**;

**proc** **factor** data=factor method=principal corr msa heywood priors=one simple

outstat=factor1 score plots=(scree) rotate=varimax;

var &disease;

**run**;

**proc** **score** data=factor score=factor1 out=score;

var &disease;

**run**;

**proc** **univariate** data=score;

var factor1-factor6;

**run**;

**proc** **freq** data=all; table mm; **run**;

**proc** **univariate** data=score;

var factor1-factor6;

output out=percent pctlpts=**85** **90** **95**

pctlpre=factor1 factor2 factor3 factor4 factor5 factor6

pctlname=pct85 pct90 pct95;

**run**;

/*0-85%,85-90%,90-95%,95-100%*/

**data** score;

set score;

if factor1<=**0.7642178829** then mm1=**0**;

else if **0.7642178829**<factor1<=**1.2837571515** then mm1=**1**;

else if **1.2837571515**<factor1<=**2.1209717976** then mm1=**2**;

else mm1=**3**;

if factor2<=**0.1689856115** then mm2=**0**;

else if **0.1689856115**<factor2<**0.2150740054** then mm2=**1**;

else if **0.2150740054**<factor2<**1.6858213156** then mm2=**2**;

else mm2=**3**;

if factor3<=-**0.134535369** then mm3=**0**;

else if -**0.134535369**<factor3<=-**0.001658333** then mm3=**1**;

else if -**0.001658333**<factor3<=**0.8662762695** then mm3=**2**;

else mm3=**3**;

if factor4<=-**0.187717123** then mm4=**0**;

else if -**0.187717123** <factor4<=**0.1037710595** then mm4=**1**;

else if **0.1037710595** <factor4<=**1.581726015** then mm4=**2**;

else mm4=**3**;

if factor5<=**0.3067224595** then mm5=**0**;

else if **0.3067224595**<factor5<=**1.0318327233** then mm5=**1**;

else if **1.0318327233**<factor5<=**2.1853762352** then mm5=**2**;

else mm5=**3**;

if factor6<=**0.0176337861** then mm6=**0**;

else if **0.0176337861**<factor6<=**0.0411782414** then mm6=**1**;

else if **0.0411782414**<factor6<=**0.5649673032** then mm6=**2**;

else mm6=**3**;

**run**;

**proc** **freq** data=score; tables mm1-mm6; **run**;

**data** pattern_factor; set score; keep n_eid mm1-mm6; **run**;

**data** all;

merge all pattern_factor;

by n_eid;

**run**;

/*Association of birth weight and childhood body size with mm patterns from exploratory factor analysis */

**proc** **freq** data=all; table mm1 mm2 mm3 mm4 mm5 mm6; **run**;

**proc** **freq** data=all; table mm1*bw_c mm2*bw_c mm3*bw_c mm4*bw_c mm5*bw_c mm6*bw_c; **run**;

**proc** **freq** data=all; table mm1*child_obe mm2*child_obe mm3*child_obe mm4*child_obe mm5*child_obe mm6*child_obe; **run**;

/*Pattern 1*/

**proc** **logistic** data=all;

class bw_c(ref='0') child_obe(ref='0') bmic(ref='0') ethnic(ref='0') tdic(ref='0') edu(ref='0')smoke(ref='0') drink(ref='0') act(ref='0') fafruit(ref='0') faveg(ref='0') m_smoke(ref='0') breast(ref='0');

model mm1(ref='0')=bw_c child_obe bmic sex ethnic tdic edu

smoke drink act fafruit faveg m_smoke breast endage/link=glogit;

**run**;

/*Pattern 2*/

**proc** **logistic** data=all;

class bw_c(ref='0') child_obe(ref='0') bmic(ref='0') ethnic(ref='0') tdic(ref='0') edu(ref='0') smoke(ref='0') drink(ref='0') act(ref='0') fafruit(ref='0') faveg(ref='0') m_smoke(ref='0') breast(ref='0');

model mm2(ref='0')=bw_c child_obe bmic sex ethnic tdic edu

smoke drink act fafruit faveg m_smoke breast endage/link=glogit;

**run**;

/*Pattern 3*/

**proc** **logistic** data=all;

class bw_c(ref='0') child_obe(ref='0') bmic(ref='0') ethnic(ref='0') tdic(ref='0') edu(ref='0') smoke(ref='0') drink(ref='0') act(ref='0') fafruit(ref='0') faveg(ref='0') m_smoke(ref='0') breast(ref='0');

model mm3(ref='0')=bw_c child_obe bmic sex ethnic tdic edu

smoke drink act fafruit faveg m_smoke breast endage/link=glogit;

**run**;

/*Pattern 4*/

**proc** **logistic** data=all;

class bw_c(ref='0') child_obe(ref='0') bmic(ref='0') ethnic(ref='0') tdic(ref='0') edu(ref='0') smoke(ref='0') drink(ref='0') act(ref='0') fafruit(ref='0') faveg(ref='0') m_smoke(ref='0') breast(ref='0');

model mm4(ref='0')=bw_c child_obe bmic sex ethnic tdic edu

smoke drink act fafruit faveg m_smoke breast endage/link=glogit;

**run**;

/*Pattern 5*/

**proc** **logistic** data=all;

class bw_c(ref='0') child_obe(ref='0') bmic(ref='0') ethnic(ref='0') tdic(ref='0') edu(ref='0') smoke(ref='0') drink(ref='0') act(ref='0') fafruit(ref='0') faveg(ref='0') m_smoke(ref='0') breast(ref='0');

model mm5(ref='0')=bw_c child_obe bmic sex ethnic tdic edu

smoke drink act fafruit faveg m_smoke breast endage/link=glogit;

**run**;

/*Pattern 6*/

**proc** **logistic** data=all;

class bw_c(ref='0') child_obe(ref='0') bmic(ref='0') ethnic(ref='0') tdic(ref='0') edu(ref='0') smoke(ref='0') drink(ref='0') act(ref='0') fafruit(ref='0') faveg(ref='0') m_smoke(ref='0') breast(ref='0');

model mm6(ref='0')=bw_c child_obe bmic sex ethnic tdic edu

smoke drink act fafruit faveg m_smoke breast endage/link=glogit;

**run**;

/*Code for multiple imputation*/

%let impute_var = ethnic edu smoke drink act m_smoke breast;

%let non_impute_var = bw_c child_obe bmic sex age_base tdic fafruit faveg mm;

**proc** **mi** data = impute nimpute = **0**;

var &impute_var &non_impute_var;

**run**;

**proc** **mi** data = impute

nimpute = **5** seed = **17** out = mi_dat;

class ethnic edu smoke drink act m_smoke breast sex;

fcs

regression(age_base /details)

discrim(ethnic edu smoke drink act m_smoke breast sex /details classeffect = include);

var ethnic edu smoke drink act m_smoke breast sex age_base;

**run**;

/*model 1*/

**proc** **phreg** data=mi_dat;

by _imputation_;

class bw_c(ref='0') child_obe(ref='0');

model mmage*mm(**0**)=bw_c child_obe bmic/risklimits;

ods output ParameterEstimates=es;

**run**;

**proc** **sort** data=es;

by parameter ClassVal0 _Imputation_;

**run**;

**proc** **mianalyze** data=es;

by parameter ClassVal0;

modeleffects estimate;

stderr StdErr;

ods output ParameterEstimates=mies;

**run**;

**data** hr;

set mies;

lghr=estimate;

hr=exp(estimate);

lcl=exp(LCLMean);

ucl=exp(UCLMean);

keep parameter ClassVal0 hr lcl ucl probt;

**run**;

/*model 2*/

**proc** **phreg** data=mi_dat;

by _imputation_;

class bw_c(ref='0') child_obe(ref='0') bmic(ref='0') ethnic(ref='0') tdic(ref='0') edu(ref='0');

model mmage*mm(**0**)=bw_c child_obe bmic sex age_base ethnic tdic edu/risklimits;

ods output ParameterEstimates=es;

**run**;

**proc** **sort** data=es;

by parameter ClassVal0 _Imputation_;

**run**;

**proc** **mianalyze** data=es;

by parameter ClassVal0;

modeleffects estimate;

stderr StdErr;

ods output ParameterEstimates=mies;

**run**;

**data** hr;

set mies;

lghr=estimate;

hr=exp(estimate);

lcl=exp(LCLMean);

ucl=exp(UCLMean);

keep parameter ClassVal0 hr lcl ucl probt;

**run**;

/*model 3*/

**proc** **phreg** data=mi_dat;

by _imputation_;

class bw_c(ref='0') child_obe(ref='0') bmic(ref='0') ethnic(ref='0') tdic(ref='0') edu(ref='0')

smoke(ref='0') drink(ref='0') act(ref='0') fafruit(ref='0') faveg(ref='0');

model mmage*mm(**0**)=bw_c child_obe bmic sex age_base ethnic tdic edu

smoke drink act fafruit faveg/risklimits;

ods output ParameterEstimates=es;

**run**;

**proc** **sort** data=es;

by parameter ClassVal0 _Imputation_;

**run**;

**proc** **mianalyze** data=es;

by parameter ClassVal0;

modeleffects estimate;

stderr StdErr;

ods output ParameterEstimates=mies;

**run**;

**data** hr;

set mies;

lghr=estimate;

hr=exp(estimate);

lcl=exp(LCLMean);

ucl=exp(UCLMean);

keep parameter ClassVal0 hr lcl ucl probt;

**run**;

/*model 4*/

**proc** **phreg** data=mi_dat;

by _imputation_;

class bw_c(ref='0') child_obe(ref='0') bmic(ref='0') ethnic(ref='0') tdic(ref='0') edu(ref='0')

smoke(ref='0') drink(ref='0') act(ref='0') fafruit(ref='0') faveg(ref='0') m_smoke(ref='0') breast(ref='0');

model mmage*mm(**0**)=bw_c child_obe bmic sex age_base ethnic tdic edu

smoke drink act fafruit faveg m_smoke breast/risklimits;

ods output ParameterEstimates=es;

**run**;

**proc** **sort** data=es;

by parameter ClassVal0 _Imputation_;

**run**;

**proc** **mianalyze** data=es;

by parameter ClassVal0;

modeleffects estimate;

stderr stderr;

ods output ParameterEstimates=mies;

**run**;

**data** hr;

set mies;

lghr=estimate;

hr=exp(estimate);

lcl=exp(LCLMean);

ucl=exp(UCLMean);

keep parameter ClassVal0 hr lcl ucl probt;

**run**;
