# Supplementary material for: Association of birth and childhood weight with risk of chronic diseases and multimorbidity in adulthood
Source: Commun Med (Lond). 2023 Jul 31;3:105. doi: 10.1038/s43856-023-00335-4 (PMC10390459; doi:10.1038/s43856-023-00335-4)
Supplement: Supplementary file 7 — Reporting Summary [file 43856_2023_335_MOESM7_ESM.pdf]

nature portfolio

Corresponding author(s): Xiaolin Xu

Last updated by author(s): Jul 18, 2023

Reporting Summary

Nature Portfolio wishes to improve the reproducibility of the work that we publish. This form provides structure for consistency and transparency in reporting. For further information on Nature Portfolio policies, see our Editorial Policies and the Editorial Policy Checklist.

Statistics

For all statistical analyses, confirm that the following items are present in the figure legend, table legend, main text, or Methods section.

n/a	Confirmed	
☐	☒	The exact sample size (*n*) for each experimental group/condition, given as a discrete number and unit of measurement
☒	☐	A statement on whether measurements were taken from distinct samples or whether the same sample was measured repeatedly
☐	☒	The statistical test(s) used AND whether they are one- or two-sided<br>*Only common tests should be described solely by name; describe more complex techniques in the Methods section.*
☐	☒	A description of all covariates tested
☐	☒	A description of any assumptions or corrections, such as tests of normality and adjustment for multiple comparisons
☐	☒	A full description of the statistical parameters including central tendency (e.g. means) or other basic estimates (e.g. regression coefficient) AND variation (e.g. standard deviation) or associated estimates of uncertainty (e.g. confidence intervals)
☐	☒	For null hypothesis testing, the test statistic (e.g. *F*, *t*, *r*) with confidence intervals, effect sizes, degrees of freedom and *P* value noted<br>*Give P values as exact values whenever suitable.*
☒	☐	For Bayesian analysis, information on the choice of priors and Markov chain Monte Carlo settings
☒	☐	For hierarchical and complex designs, identification of the appropriate level for tests and full reporting of outcomes
☒	☐	Estimates of effect sizes (e.g. Cohen's *d*, Pearson's *r*), indicating how they were calculated

Our web collection on statistics for biologists contains articles on many of the points above.

Software and code

Policy information about availability of computer code

Data collection	No software was used to collect data.
Data analysis	Statistical analyses were performed using SAS (version 9.4, SAS Institute Inc., NC, USA) and R (version 4.1.2, R Foundation for Statistical Computing).

For manuscripts utilizing custom algorithms or software that are central to the research but not yet described in published literature, software must be made available to editors and reviewers. We strongly encourage code deposition in a community repository (e.g. GitHub). See the Nature Portfolio guidelines for submitting code & software for further information.

Data

Policy information about availability of data

All manuscripts must include a data availability statement. This statement should provide the following information, where applicable:

- Accession codes, unique identifiers, or web links for publicly available datasets
- A description of any restrictions on data availability
- For clinical datasets or third party data, please ensure that the statement adheres to our policy

This research was conducted using the UK Biobank study under Application Number 66354. The original data for the study are available on the UK Biobank website: https://www.ukbiobank.ac.uk/.

Human research participants

Policy information about studies involving human research participants and Sex and Gender in Research.

Reporting on sex and gender	There is no sex and gender-based analyses been performed in this study, given that the objective of this study is to assess the association of birth weight, childhood body size, and their longitudinal changes with the lifetime risk of chronic diseases and multimorbidity in the general population.
Population characteristics	We included 246,495 participants with self-reported information on their birth weight and childhood body size in the UK Biobank. The mean baseline age was 55.2 years, and 61.1% participants were female. The proportion of participants reported low birth weight and high birth weight was 8.8% and 14%, respectively. A total of 32.2% participants reported being slimmer and 16.6% reported being plumper at 10 years old. Among these participants, 23.2% of them developed multimorbidity.
Recruitment	Participants in this study were recruited in the UK Biobank baseline assessment between 2006 and 2010. The UK Biobank participants were healthier and more affluent than the average UK population, therefore, the included participants may not be representative of the general UK population.
Ethics oversight	Participants provided informed consent. The UK Biobank has ethical approval from the NHS National Research Ethics Service (16/NW/0274). This is a secondary analysis of UK Biobank which is exempted from Institutional Review Board.

Note that full information on the approval of the study protocol must also be provided in the manuscript.

Field-specific reporting

Please select the one below that is the best fit for your research. If you are not sure, read the appropriate sections before making your selection.

☒ Life sciences ☐ Behavioural & social sciences ☐ Ecological, evolutionary & environmental sciences

For a reference copy of the document with all sections, see nature.com/documents/nr-reporting-summary-flat.pdf

Life sciences study design

All studies must disclose on these points even when the disclosure is negative.

Sample size	184093
Data exclusions	Participants were excluded if they (1): had no information on birth weight, childhood body size, and adulthood body mass index; (2) reported being part of multiple births; (3) lost or died during follow-up.
Replication	Findings of this study are generated by the statistical analyses described in the methodology. All data required for the analysis are publicly available in the UK Biobank website. The programming code is available upon reasonable request. The analyses were run repeatedly and sensitivity analyses were conducted to verify the robustness of the main results.
Randomization	Not applicable
Blinding	Not applicable

Reporting for specific materials, systems and methods

We require information from authors about some types of materials, experimental systems and methods used in many studies. Here, indicate whether each material, system or method listed is relevant to your study. If you are not sure if a list item applies to your research, read the appropriate section before selecting a response.

Materials & experimental systems

n/a	Involved in the study	
☒	☐	Antibodies
☒	☐	Eukaryotic cell lines
☒	☐	Palaeontology and archaeology
☒	☐	Animals and other organisms
☒	☐	Clinical data
☒	☐	Dual use research of concern

Methods

n/a	Involved in the study	
☒	☐	ChIP-seq
☒	☐	Flow cytometry
☒	☐	MRI-based neuroimaging
